# Genome-Wide Dynamic Profiling of Histone Methylation during Nuclear Transfer-Mediated Porcine Somatic Cell Reprogramming

**DOI:** 10.1371/journal.pone.0144897

**Published:** 2015-12-18

**Authors:** Zubing Cao, Yunsheng Li, Zhen Chen, Heng Wang, Meiling Zhang, Naru Zhou, Ronghua Wu, Yinghui Ling, Fugui Fang, Ning Li, Yunhai Zhang

**Affiliations:** 1 Anhui Provincial Laboratory of Local Livestock and Poultry Genetical Resource Conservation and Breeding, College of Animal Science and Technology, Anhui Agricultural University, Hefei City, Anhui Province, China; 2 State Key Laboratory for Agrobiotechnology, College of Biological Science, China Agricultural University, Haidian District, Beijing, China; Institute of Zoology, Chinese Academy of Sciences, CHINA

## Abstract

The low full-term developmental efficiency of porcine somatic cell nuclear transfer (SCNT) embryos is mainly attributed to imperfect epigenetic reprogramming in the early embryos. However, dynamic expression patterns of histone methylation involved in epigenetic reprogramming progression during porcine SCNT embryo early development remain to be unknown. In this study, we characterized and compared the expression patterns of multiple histone methylation markers including transcriptionally repressive (H3K9me2, H3K9me3, H3K27me2, H3K27me3, H4K20me2 and H4K20me3) and active modifications (H3K4me2, H3K4me3, H3K36me2, H3K36me3, H3K79me2 and H3K79me3) in SCNT early embryos from different developmental stages with that from in vitro fertilization (IVF) counterparts. We found that the expression level of H3K9me2, H3K9me3 and H4K20me3 of SCNT embryos from 1-cell to 4-cell stages was significantly higher than that in the IVF embryos. We also detected a symmetric distribution pattern of H3K9me2 between inner cell mass (ICM) and trophectoderm (TE) in SCNT blastocysts. The expression level of H3K9me2 in both lineages from SCNT expanded blastocyst onwards was significantly higher than that in IVF counterparts. The expression level of H4K20me2 was significantly lower in SCNT embryos from morula to blastocyst stage compared with IVF embryos. However, no aberrant dynamic reprogramming of H3K27me2/3 occurred during early developmental stages of SCNT embryos. The expression of H3K4me3 was higher in SCNT embryos at 4-cell stage than that of IVF embryos. H3K4me2 expression in SCNT embryos from 8-cell stage to blastocyst stage was lower than that in the IVF embryos. Dynamic patterns of other active histone methylation markers were similar between SCNT and IVF embryos. Taken together, histone methylation exhibited developmentally stage-specific abnormal expression patterns in porcine SCNT early embryos.

## Introduction

Somatic cell nuclear transfer (SCNT) is a reproductive biotechnology, which can reprogram highly differentiated somatic cells into totipotent embryos and further develop into individual animals. Studies in different mammalian species demonstrated abnormal reprogramming of epigenetic modification in the donor cell genome is the most important causes of low cloning efficiency [1, 2]. Donor cell nuclei contain highly specific DNA methylation and epigenetic information of histone modification. Somatic cell cloned embryos require global genome dynamic epigenetic information reprogramming to obtain the developmental capacities, which is similar with the process of normal embryos during early embryonic development. Abnormal DNA methylation reprogramming in somatic cell cloned animals or embryos was reported in mouse[3], cattle[4–7], pig[8, 9], rabbit[10] and sheep[11], but reprogramming mechanisms of histone modifications during the early development process of SCNT embryos are still poorly understood[12, 13]. Histone modification is an important epigenetic modification for regulating gene expression which includes acetylation, methylation, phosphorylation, ubiquitination [14]. Different amino acid residuals and variable degree of methylation may result in different biological functions. In general, methylation of H3K4, H3K36 and H3K79 is related with activation of gene transcription, whereas H3K9, H3K27 and H4K20 methylation mainly inhibit gene expression. Histone methylation may also play an important role in the somatic cell reprogramming, although previous studies have found that high levels of histone acetylation can improve the developmental efficiency of somatic cloned embryos [15–17]. Recent studies found that abnormal reprogramming of histone methylation in early developmental procession of SCNT embryo, such as abnormal reprogramming of H3K9me3 [18] and H3K27me3 [19] in mouse, H3K9me3 [6] in bovine, H3K9me2 in sheep [20], and H3K36me3 [13] and H3K4me3 [12] in pig cloned embryos.

Given the general lack of evidences on histone methylation reprogramming during early development of porcine SCNT embryos, we investigated dynamic reprogramming of histone methylation modifications, such as di- and tri-methylation of H3 and H4 lysine residues, which probably affect the development of SCNT embryos. We found aberrant epigenetically targets of histone methylation reprogramming during development of SCNT embryos as compared with IVF embryos. These results indicated modifying aberrant targets of histone methylation reprogramming at early stage might be a promising strategy to improve cloning efficiency in pig.

## Materials and Methods

All chemicals were purchased from Sigma-Aldrich (St. Louis, MO), unless stated otherwise.

### Animals

All animal experiments were approved by the Animal Care and Use Committee of Anhui Agricultural University. All procedures were carried out in strict accordance with the recommendations made in the Guide for the Care and Use of Laboratory Animals of the National Veterinary and Quarantine Service.

### In Vitro Maturation

This experiment was performed as described previously [16]. Briefly, ovaries were taken from crossbred gilts and sows (Landrace×Large White×Duroc) at the Hefei Wanrun Slaughterhouse, Anhui, China. These were placed at 28–35°C in a physiological saline solution containing penicillin (10 IU/ml) and streptomycin sulfate (10 IU/ml), and were taken to the laboratory within two hours and washed with physiological saline solution. Immediately upon arrival, ovarian follicles at 3−6 mm in diameter were aspirated using sterile 10 ml syringes with 18 G needles. The aspirated follicular fluid was slowly injected into a 38.5°C pre-warmed 15 ml centrifuge tube to sediment the cumulus-oocyte complexes (COCs). After approximately 15 min of sedimentation, a clear interface was visible. Following the removal of the supernatant, the cell pellets were diluted with oocyte-washing medium (DPBS plus 0.01% polyvinyl alcohol (PVA)) and gently pipetted up and down. Using a stereomicroscope, the COCs with more than two layers of compact cumulus investment and a dense, homogeneous cytoplasm were rapidly selected. They were washed three times with oocyte-washing medium (0.01% PVA in DPBS), then three more times with *in vitro* maturation (IVM) medium (TCM-199 supplemented with 15% FBS, 10 ng/ml EGF, 10% porcine follicular fluid, 10 IU/ml of eCG, 5 IU/ml of hCG, 0.8 mM L-glutamine and 0.05 mg/ml gentamicin). Subsequently, 50 of the washed COCs were matured in 400 μl IVM medium at 38.5°C with 5% CO_2,_ and saturated humidity for 42–44 h. COCs were treated with DPBS without Ca^2+^ and Mg^2+^ (Gibco) containing 1 mg/ml hyaluronidase to remove the surrounding cumulus cells. Finally, the oocytes with clear perivitelline spaces, intact cell membranes, and extruded first polar bodies (pb1) were selected for use.

### In Vitro Fertilization

Fresh semen was washed three times with DPBS supplemented with 0.1% BSA, 75 μg/ml penicillin G and 50 μg/ml streptomycin, and centrifugation at 100 g for 3 min. After removing the supernatant, spermatozoa pellets were resuspended with fertilization medium (mTBM) containing 2 mg/ml BSA (fraction V) plus 2 mM caffeine [21] and diluted to 0.5×10^6^−1.5×10^6^ sperm/ml. Groups of 25 denuded oocytes were washed three times in fertilization medium and then transferred to 100 μl fertilization medium covered with paraffin oil. Fifty microliters of diluted spermatozoa (around 0.5× 10^6^−1.5 × 10^6^ sperm/ml) was added to 100 μl fertilization medium containing oocytes to give a final sperm concentration of 1.5× 10^5^−5.0 × 10^5^ sperm/ml. The oocytes were co-incubated with sperm for 5 h at 38.5°C with 5% CO_2_ in air.

### Construction, Fusion, and Activation of SCNT Embryos

#### Embryo Reconstruction

Matured oocytes and donor cells were simultaneously transferred into micromanipulation medium droplets. After equilibration at 38.5°C with 5% CO_2_ and saturated humidity for 10−15 min, the oocytes were held with a holding pipette (inner diameter 25−35 μm, outer diameter 100−120 μm) under an inverted microscope (Olympus IX71, Japan) equipped with a micromanipulator (Narishige, Japan) and a warmed stage (Tokihai, Japan). The first polar body (pb1) was adjusted to the 1 o’clock position using an enucleation/microinjection needle with a 15−25 μm inner diameter. Subsequently, from the 3 o’clock position, the microinjection needle was inserted to draw the first polar body together with 10% −20% of the adjacent cytoplasm, which potentially contained the oocyte nucleus. Porcine fetal fibroblast cells, which were 15−20 μm in diameter, highly refractive, and rounded to a smooth shape, were selected for transfer into the perivitelline space from the enucleating cut. Following this micromanipulation, the reconstructed oocytes composed of a donor cell and oocyte cytoplasm were transferred into a T2 (TCM199 + 2% FBS) droplet and placed in an incubator for 30 min at 38.5°C with 5% CO_2_ and saturated humidity for a recovery period.

#### Fusion and Activation of Reconstructed Embryos

After the 30 min recovery period in the T2 droplet, the reconstructed oocytes were transferred into fusion medium (0.3 M mannitol supplemented with 0.05 mM CaCl_2_, 0.1 mM MgSO_4_ and 0.01% polyvinyl alcohol) in batches of 10−20 reconstructed oocytes to equilibrate for two minutes. Following three washes with fusion medium, each batch was placed in cell fusion chamber and the reconstructed oocytes were spread with fusion medium. By moving the reconstructed oocytes with pulled fine glass needle, the contact surfaces of the donor cell and the recipient oocyte were oriented parallel to the electrodes. With CF-150B cell fusion instrument (BLS, Hungary), single direct current (DC) pulse of 1.56 kV/cm for 100 μs was administered to induce cell fusion and activation. Subsequently, couplets were washed three times with porcine zygote medium 3 (PZM-3)[22] and immediately transferred into chemically assisted activation solution (PZM-3 plus 10 μg/ml Cycloheximide (CHX) and 10 μg/ml Cytochalasin B (CB)) and covered with mineral oil. After 4 h in an incubator at 38.5°C with 5% CO_2_ and saturated humidity, fusion results were determined using a stereomicroscope.

### In Vitro Culture

*In vitro* fertilized or chemical post-activated embryos (SCNT) were washed three times with PZM-3. Approximately 50 embryos were cultured in 4-well dishes containing 400 μl PZM-3. They were collected at different time points to obtain embryos at different developmental stages. Embryo culture conditions were 38.5°C, 5% CO_2_, 95% air, and 100% humidity.

### Immunofluorescence Staining and Quantification Analysis

Embryos used for immunofluorescence staining were treated with pronase solution (0.5% pronase in DPBS) to remove the outer zona pellucida. The embryos were fixed for 15 min in 4% paraformaldehyde (PFA) in PBS, washed three times for 15 min. The fixed embryos were permeabilized with 0.5% Triton X-100 in DPBS for 30 min at room temperature (RT), then washed briefly with DPBS containing 0.3% PVP. Then embryos were blocked with 1% BSA in DPBS overnight at 4°C. Embryos were subsequently incubated in blocking buffer containing primary antibodies against different histone methylation sites (S1 Table) at 37°C for 1 h, washed 12 times in 60 min with 0.3% PVP in DPBS and incubated for 1 h with secondary antibodies, including goat anti-Rabbit IgG conjugated with Alexa Fluor 488 (Molecular probe, A11008, 1:200) or goat anti-mouse IgG conjugated with Alexa Fluor 488 (Molecular probe, A11029, 1:200) in the dark at 37°C. Finally, embryos were washed 12 times in 60 min with 0.3% PVP in DPBS and counterstained with 1 μg/ml PI (Sigma, P4170) for 10 min. Finally, embryos were washed several times and mounted on glass slides with a small drop of Vectashield (VectorLab) mounting medium, then covered by a glass coverslip and assessed using confocal laser scanning microscopy (Olympus, FluoView1000). Negative control staining was also performed. In these cases, the primary antibody was replaced with the blocking buffer. IVF and SCNT embryos (n = 10–15) from different time points or developmental stages were used for every immunostaining procedures, and three replicates were conducted for each experiment.

The signal intensity for all histone markers in oocytes and embryos was analyzed as described previously [23]. Briefly, nuclei of blastomeres were identified by DAPI staining using the 40× objective lens. Using the same field of view and objective, fluorescence staining images of histone methylation marker were captured. Quantification analysis of histone methylation markers in nuclei or cytoplasmic areas was performed using Image J software. The border around the nuclei was manually delineated according to DNA staining. In addition, at least three different cytoplasmic areas were delineated for normalization to background. The average pixel intensity of the nuclear areas was calculated by Image J, and then normalized by dividing by the average pixel intensity of the background areas [24].

### Statistical Analysis

All experiments were repeated at least three times. Unless noted otherwise, experimental data are presented as mean ± S.E.M. values. Differences in the levels of global histone methylation patterns in embryos were performed using one-way analysis of variance (ANOVA) with SPSS software (version 11.5). *P<* 0.05 was considered statistically significant.

## Results

### Dynamic Reprogramming of H3K4me2/3 during Early Development of IVF and SCNT Embryos

**Fig 1:**
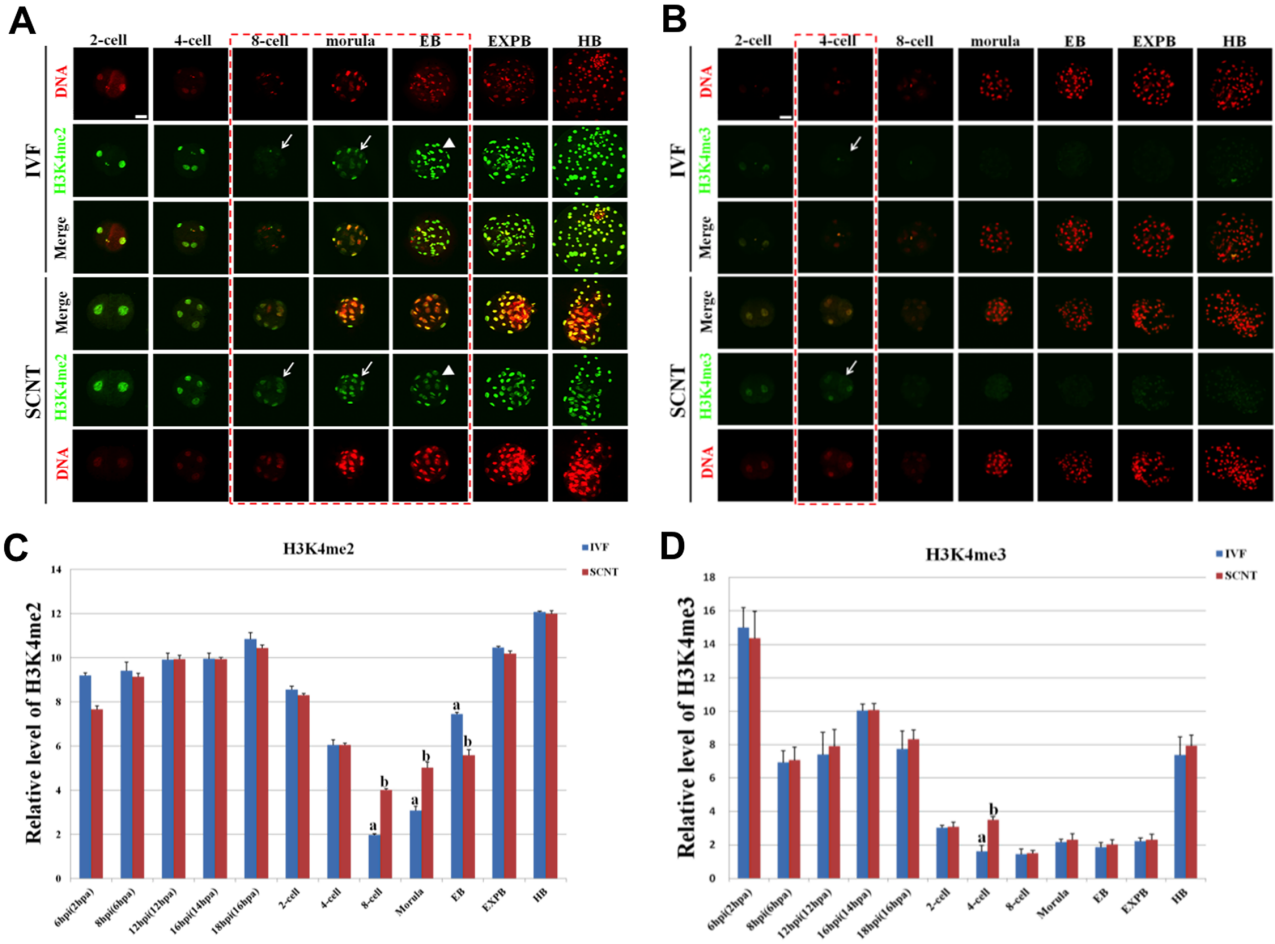
Dynamic patterns of H3K4me2/3 during porcine IVF and SCNT cleavage-stages embryo development. **(A)** Representative images of IVF and SCNT embryos at different developmental stages immunostained with an anti-H3K4me2 antibody. Antibody was localized with an Alexa Flour 488-conjugated secondary antibody (green). DNA was stained with propidium iodide (red). Middle panels showed the merged images (yellow) between H3K4me2 signal (green) and DNA staining (red). Red dash line marks the developmental stages with different H3K4me2 signal intensity between IVF and SCNT embryos. Arrow denotes H3K4me2 signal intensity was abnormally higher in SCNT embryos than that in IVF counterparts. Arrowhead means H3K4me2 signal intensity was abnormally lower in SCNT embryos than that in IVF counterparts. **(B)** Representative images of IVF and SCNT embryos at different developmental stages immunostained with an anti-H3K4me3 antibody. Arrow denotes H3K4me3 signal intensity was abnormally higher in SCNT embryos than that in IVF counterparts. Scale bar = 50 μm. **(C)** Quantification of H3K4me2 intensity between IVF and SCNT early embryos. (D) Quantification of H3K4me3 intensity between IVF and SCNT early embryos. Blue bars denote IVF group, red bars represent SCNT group. Values are mean ± S.E.M. Different letters (a-b) on the bars indicate a statistically significant difference between IVF and SCNT groups (*p* < 0.05).

H3K4me2/3 showed symmetric distribution patterns between female and male pronuclei in IVF embryos (S1A and S1B Fig). The signal intensity of H3K4me2 during preimplantation development of IVF embryo gradually decreased to the minimum from 1-cell stage to 8-cell stage, and then reappeared at morula stage, followed by a significant increase throughout the blastocyst stages (Fig 1A). Like the dynamic patterns of H3K4me2, the signal intensity of H3K4me3 during preimplantation development of IVF embryo steadily decreased to the undetectable level from 1-cell stage to 4-cell stage, then retained the lowest level until the expanded blastocyst stage, followed by a significant increase at the hatched blastocyst stage (Fig 1B).

The signal intensity of H3K4me2 in SCNT embryos at 8-cell and morula stage was significantly higher than that in IVF counterparts (*p* < 0.05), whereas the signal intensity of H3K4me2 in SCNT early blastocysts was significantly lower than that in IVF counterparts (*p* < 0.05) (Fig 1C). In addition, the signal intensity of H3K4me3 in SCNT embryos at 4-cell stage was significantly higher than that in IVF counterparts (*p* < 0.05) (Fig 1D). No significant difference was detected at the other developmental periods (pronuclear and cleavage stages) between SCNT and IVF embryos.

### Dynamic Patterns of H3K9me2/3 during Early Development of IVF and SCNT Embryos

**Fig 2:**
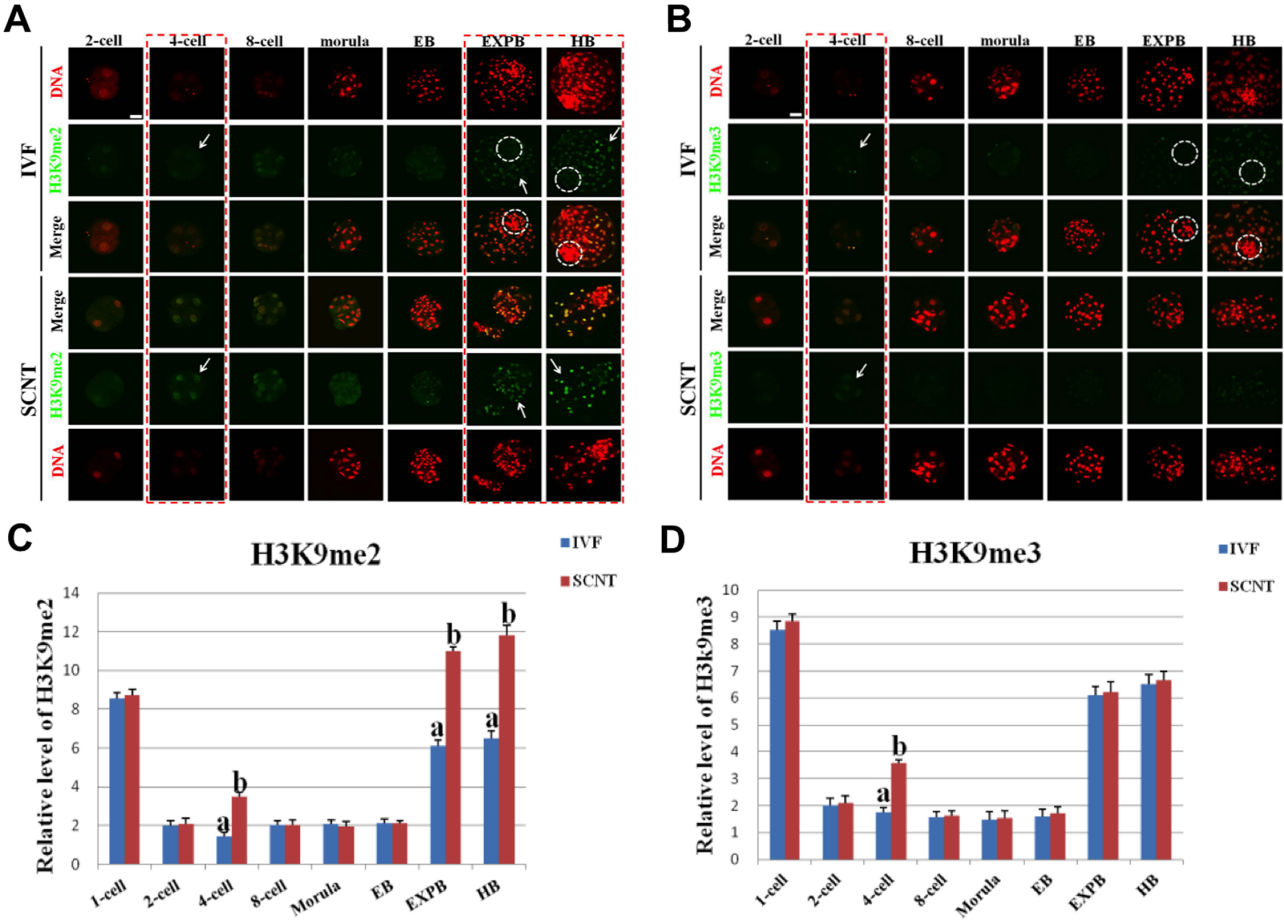
Dynamic patterns of H3K9me2/3 during IVF and SCNT cleavage-stages embryo development. **(A)** Representative images of porcine IVF and SCNT embryos at different developmental stages immunostained with an anti-H3K9me2 antibody. Antibody was localized with an Alexa Flour 488-conjugated secondary antibody (green). DNA was stained with propidium iodide (red). Middle panels showed the merged images (yellow) between H3K9me2 signal (green) and DNA staining (red). Red dash line marks the developmental stages with different H3K9me2 signal intensity between IVF and SCNT embryos. Arrow denotes H3K9me2 signal intensity was abnormally higher in SCNT embryos than that in IVF counterparts. White dash circle denotes ICM. **(B)** Representative images of IVF and SCNT embryos at different developmental stages immunostained with an anti-H3K9me3 antibody. Arrow denotes H3K9me3 signal intensity was abnormally higher in SCNT embryos than that in IVF counterparts. White dash circle denotes ICM. Scale bar = 50 μm. **(C)** Quantification of H3K9me2 intensity between IVF and SCNT early embryos. (D) Quantification of H3K9me3 intensity between IVF and SCNT early embryos. Blue bars denote IVF group, red bars represent SCNT group. Values are mean ± S.E.M. Different letters (a-b) on the bars indicate a statistically significant difference between IVF and SCNT groups (*p* < 0.05).

H3K9me2/3 exhibited symmetric distribution between maternal and paternal genome in IVF pronuclear embryos (S2A Fig), and the signal intensity had no remarkable difference between IVF and SCNT pronuclear embryos (Fig 2C and 2D). The signal intensity of H3K9me2/3 during early development of IVF embryo suddenly decreased to the minimum level from 1-cell stage to 2-cell stage, then retained this pattern until the early blastocyst stage, followed by a significant increase at the expanded blastocyst stage (Fig 2A and 2B). H3K9me2/3 were exclusively expressed in ICM region of IVF blastocysts, whereas they were ectopically expressed in both ICM and TE region of SCNT blastocysts (Fig 2A and 2B). Moreover, the signal intensity of H3K9me2/3 in SCNT embryos at 4-cell stage around the time of embryonic genome activation was apparently higher than that in IVF counterparts (*p* < 0.05) (Fig 2C), and the signal intensity of H3K9me2 in TE region of SCNT expanded and hatched blastocysts was significantly higher than that IVF counterparts (*p* < 0.05) (Fig 2D). There was no significant difference in intensity of the two epigenetic modifications at the other developmental stages between SCNT and IVF embryos.

### Dynamic Changes of H3K27me2/3 during Early Development of IVF and SCNT Embryos

**Fig 3:**
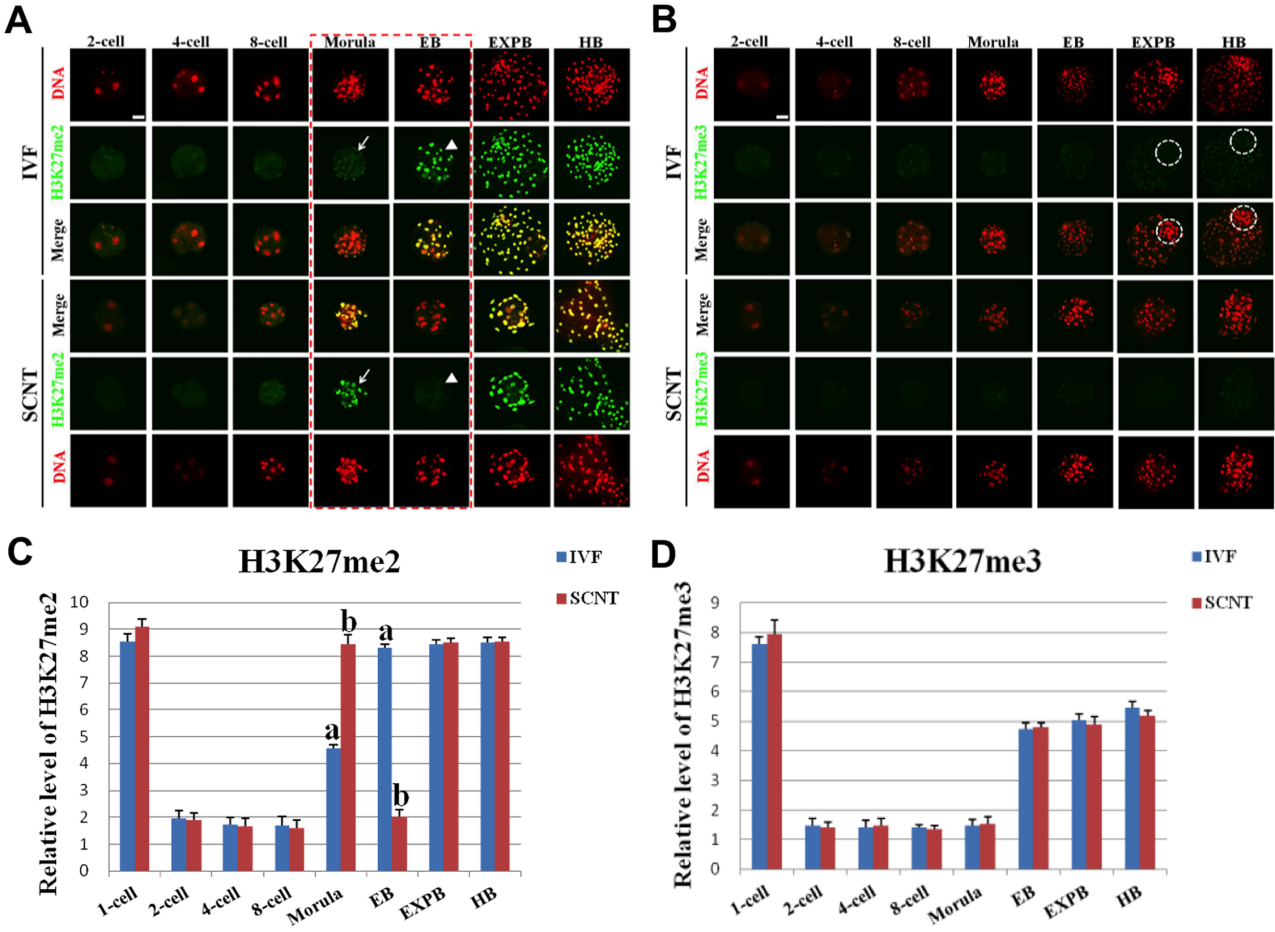
Dynamic patterns of H3K27me2/3 during IVF and SCNT cleavage-stages embryo development. **(A)** Representative images of porcine IVF and SCNT embryos at different developmental stages immunostained with an anti-H3K27me2 antibody. Antibody was localized with an Alexa Flour 488-conjugated secondary antibody (green). DNA was stained with propidium iodide (red). Middle panels showed the merged images (yellow) between H3K27me2 signal (green) and DNA staining (red). Red dash line marks the developmental stages with different H3K27me2 signal intensity between IVF and SCNT embryos. Arrow denotes H3K27me2 signal intensity was abnormally higher in SCNT embryos than that in IVF counterparts. Arrowhead means H3K27me2 signal intensity was abnormally lower in SCNT embryos than that in IVF counterparts. **(B)** Representative images of IVF and SCNT embryos at different developmental stages immunostained with an anti-H3K27me3 antibody. White dash circle denotes ICM. Scale bar = 50 μm. **(C)** Quantification of H3K27me2 intensity between IVF and SCNT early embryos. (D) Quantification of H3K27me3 intensity between IVF and SCNT early embryos. Blue bars denote IVF group, red bars represent SCNT group. Values are mean ± S.E.M. Different letters (a-b) on the bars indicate a statistically significant difference between IVF and SCNT groups (*p <* 0.05).

Interestingly, H3K27me2 exhibited asymmetric distribution between female and male pronuclei in IVF embryos (S3A Fig), and the signal intensity of H3K27me2 in female pronuclei was higher compared with that in male pronuclei (Fig 3C). H3K27me3 showed uniform distribution patterns between female and male pronuclei in IVF embryos (S3A Fig). The expression levels of H3K27me2/me3 during preimplantation development of IVF embryos quickly decreased from pronuclear stage and reached the minimum at 2-cell stage, then retained the lowest level until the morula stage, and followed by a remethylation throughout the blastocyst stages (Fig 3A and 3B). The signal intensity of H3K27me2 in SCNT morula was obviously higher than that in IVF counterparts (Fig 3C). However, the signal intensity of H3K27me2 in SCNT early blastocysts was significantly lower compared with that in IVF counterparts (*p* < 0.05) (Fig 3C). There were similar in the signal intensity of H3K27me2/me3 at the other developmental stages between SCNT and IVF embryos (Fig 3C and 3D).

### Dynamic Expression of H3K36me2/3 during Early Development of IVF and SCNT Embryos

**Fig 4:**
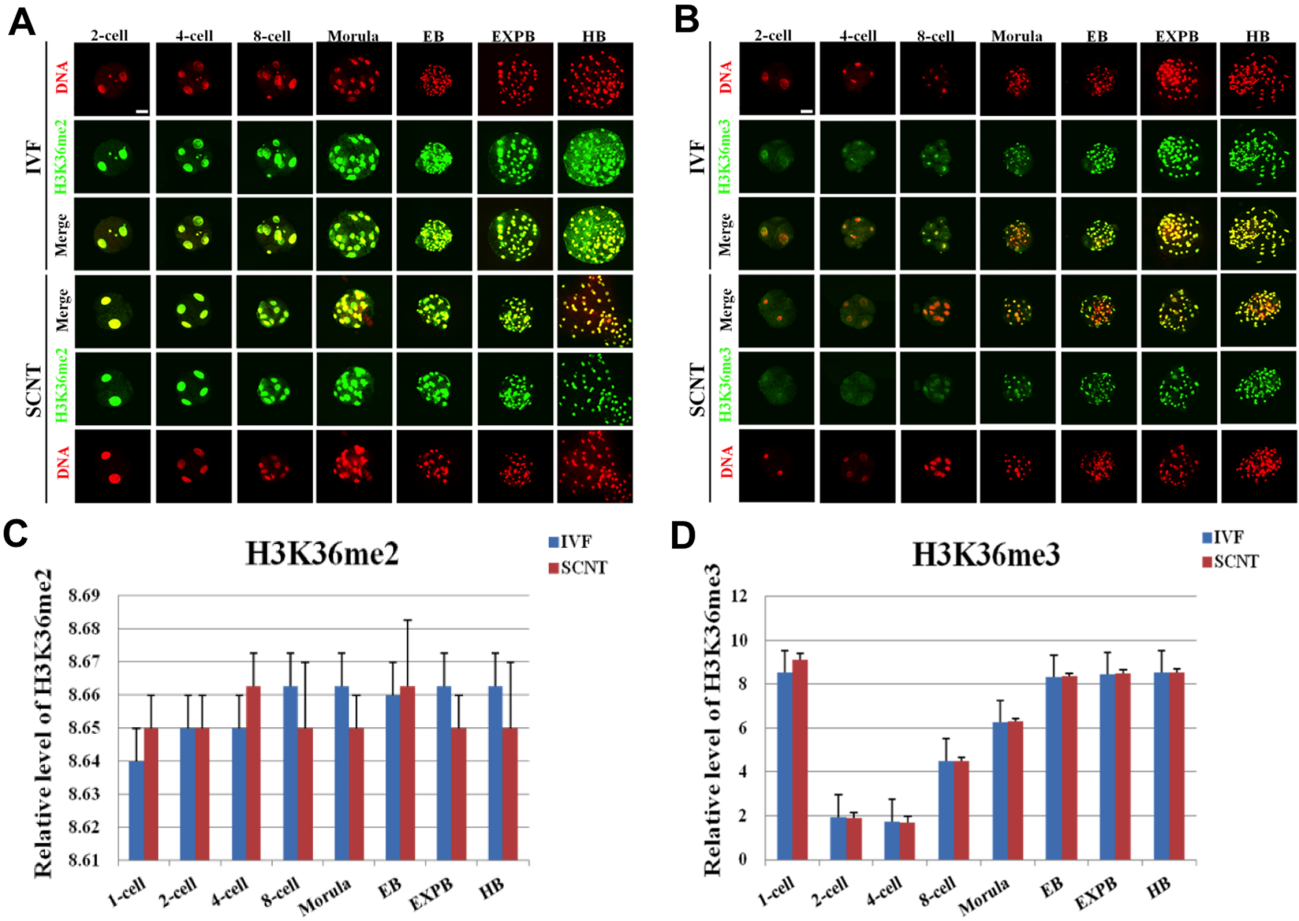
Dynamic patterns of H3K36me2/3 during IVF and SCNT cleavage-stages embryo development. **(A)** Representative images of porcine IVF and SCNT embryos at different developmental stages immunostained with an anti-H3K36me2 antibody. Antibody was localized with an Alexa Flour 488-conjugated secondary antibody (green). DNA was stained with propidium iodide (red). Middle panels showed the merged images (yellow) between H3K36me2 signal (green) and DNA staining (red). **(B)** Representative images of porcine IVF and SCNT embryos at different developmental stages immunostained with an anti-H3K36me2 antibody. Scale bar = 50 μm. **(C)** Quantification of H3K36me2 intensity between IVF and SCNT early embryos. (D) Quantification of H3K36me2 intensity between IVF and SCNT early embryos. Blue bars denote IVF group, red bars represent SCNT group. Values are mean ± S.E.M.

Symmetric distribution patterns of H3K36me2/3 were observed between female and male pronuclei in IVF embryos (S4A Fig). The signal intensity of H3K36me2 in IVF embryos invariably maintained the high expression throughout the early developmental stages (Fig 4A). The signal intensity of H3K36me3 gradually decreased from the pronuclear to 4-cell stage and followed by a stable increase from the 8-cell stage to hatched blastocyst stage (Fig 4B). However, the expression levels of H3K36me2/3 at the same developmental stages during the early development were similar between SCNT and IVF embryos (Fig 4C and 4D).

### Expression Patterns of H3K79me2/3 during Early Development of IVF and SCNT Embryos

**Fig 5:**
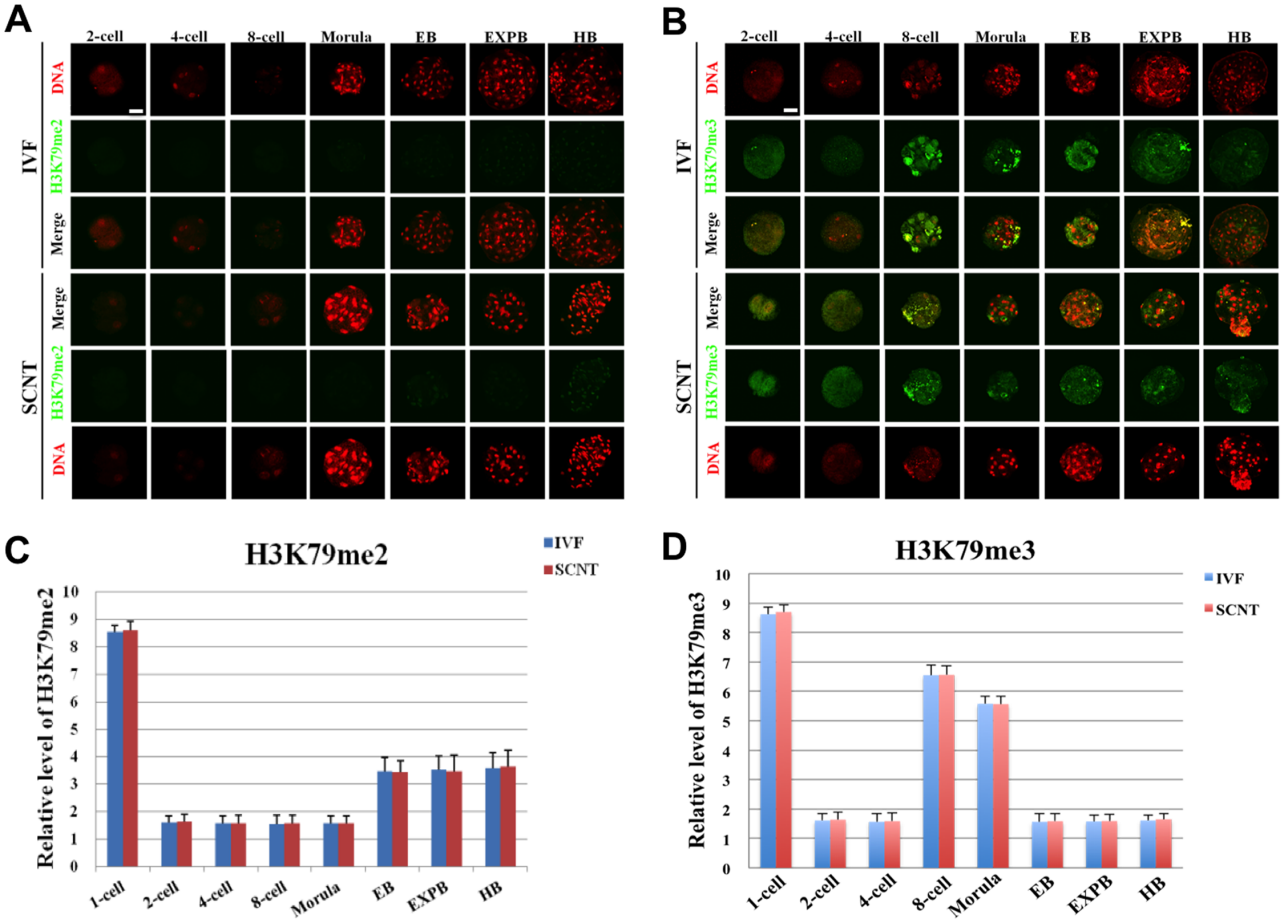
Dynamic patterns of H3K79me2/3 during IVF and SCNT cleavage-stages embryo development. **(A)** Representative images of porcine IVF and SCNT embryos at different developmental stages immunostained with an anti-H3K79me2 antibody. Antibody was localized with an Alexa Flour 488-conjugated secondary antibody (green). DNA was stained with propidium iodide (red). Middle panels showed the merged images (yellow) between H3K79me2 signal (green) and DNA staining (red). **(B)** Representative images of porcine IVF and SCNT embryos at different developmental stages immunostained with an anti-H3K79me3 antibody. Scale bar = 50 μm. **(C)** Quantification of H3K79me2 intensity between IVF and SCNT early embryos. (D) Quantification of H3K79me2 intensity between IVF and SCNT early embryos. Blue bars denote IVF group, red bars represent SCNT group. Values are mean ± S.E.M.

Porcine MII oocytes exhibited high expression of H3K79me2/3 (S5A Fig). The signal of H3K79me2 immediately disappeared after fertilization and remained undetectable until morula stage, then gradually reappeared with weak intensity throughout the blastocyst stages (Fig 5A). After fertilization, H3K79me3 signal mainly showed punctuate expression pattern in pronuclei heterochromatic regions, and decreased quickly to low level and remained such status till the 8-cell stage, then gradually reappeared with weak intensity from 8-cell onwards (S5B Fig and Fig 5B). There was no significant difference of the signal intensity of H3K79me2/3 at all the developmental stages between SCNT and IVF embryos (Fig 5C and 5D).

### Dynamic Patterns of H4K20me2/3 during Early Development of IVF and SCNT Embryos

**Fig 6:**
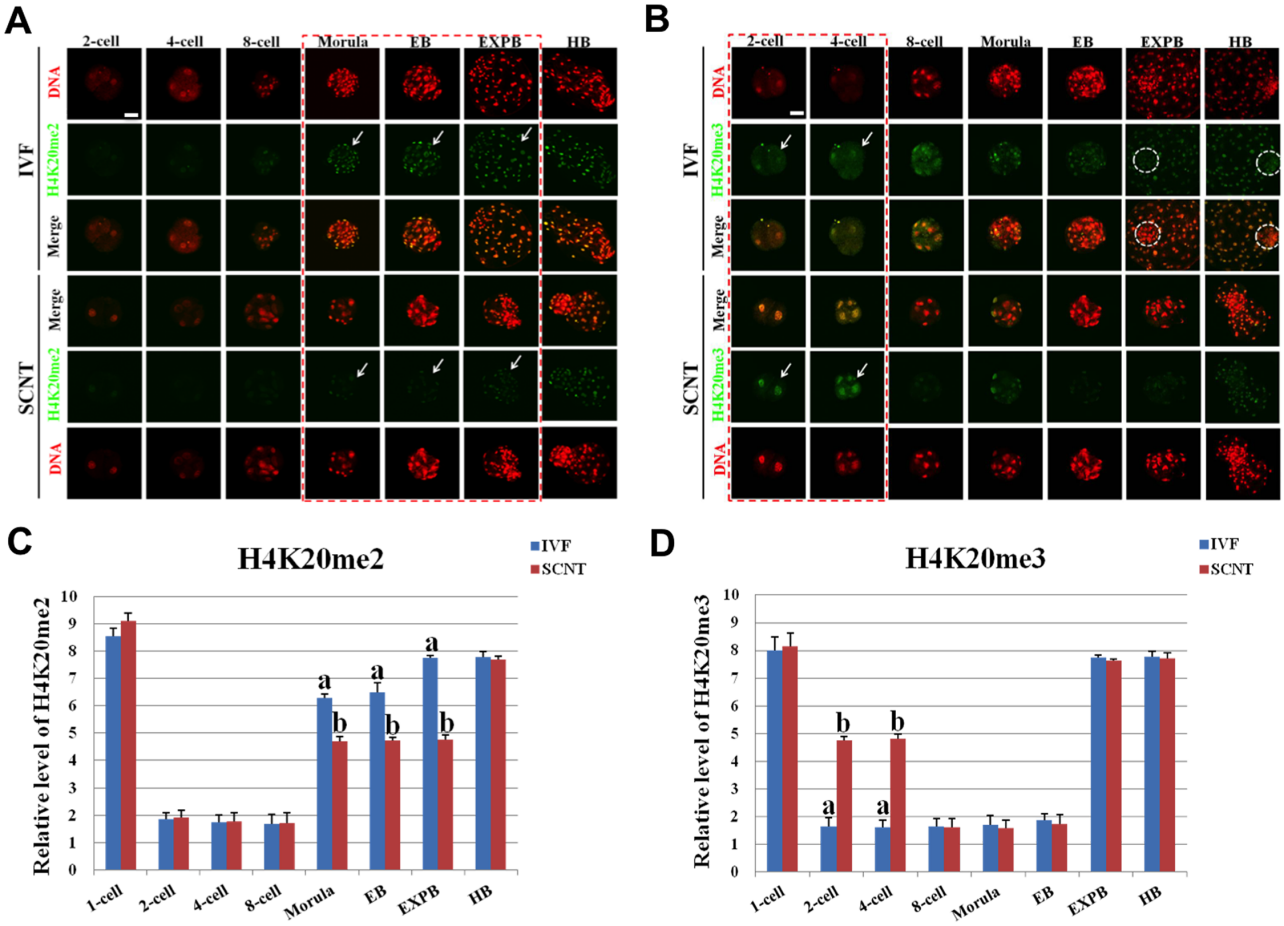
Dynamic patterns of H4K20me2/3 during IVF and SCNT cleavage-stages embryo development. **(A)** Representative images of porcine IVF and SCNT embryos at different developmental stages immunostained with an anti-H4K20me2 antibody. Antibody was localized with an Alexa Flour 488-conjugated secondary antibody (green). DNA was stained with propidium iodide (red). Middle panels showed the merged images (yellow) between H4K20me2 signal (green) and DNA staining (red). Red dash line marks the developmental stages with different H4K20me2 signal intensity between IVF and SCNT embryos. Arrow denotes H4K20me2 signal intensity was abnormally lower in SCNT embryos than that in IVF counterparts. **(B)** Representative images of porcine IVF and SCNT embryos at different developmental stages immunostained with an anti-H4K20me3 antibody. Arrow denotes H4K20me3 signal intensity was abnormally higher in SCNT embryos than that in IVF counterparts. White dash circle denotes ICM. Scale bar = 50 μm. **(C)** Quantification of H4K20me2 intensity between IVF and SCNT early embryos. (D) Quantification of H4K20me3 intensity between IVF and SCNT early embryos. Blue bars denote IVF group, red bars represent SCNT group. Values are mean ± S.E.M. Different letters (a-b) on the bars indicate a statistically significant difference between IVF and SCNT groups (*p* < 0.05).

Symmetric distribution pattern of H4K20me2/3 was also observed between maternal and paternal genome of IVF pronuclear embryos (S6A Fig). The signal intensity of H4K20me2 during early development of IVF embryo gradually decreased to minimum at 2-cell stage, then kept the lowest levels until 8-cell stage, followed by a sudden increase from morula stage to hatched blastocyst stage (Fig 6A). The signal intensity of H4K20me3 during preimplantation development of IVF embryo gradually decreased to minimum at 2-cell stage, then remained the lowest expression until early blastocyst stage, followed by a quick increase from expanded blastocyst stage to hatched blastocyst stage (Fig 6B). However, the signal intensity of H4K20me2 in SCNT embryos from morula stage to expanded blastocyst stage was significantly lower than that in IVF counterparts (*p* < 0.05) (Fig 6C). The signal intensity of H4K20me3 in SCNT embryos at 4-cell stage was significantly higher than that in IVF counterparts (*p* < 0.05) (Fig 6D). The signal intensity of H4K20me2/3 at the other developmental stages is similar between SCNT and IVF embryos.

## Discussion

In the present study, we examined epigenetic dynamics and distribution patterns of histone methylation in the process of early development of porcine IVF and SCNT embryos. We found that epigenetic information of histone methylation in IVF and SCNT embryo exhibits dynamic reprogramming patterns coincided with the porcine embryonic genome activation and lineage specification and commitment of embryonic development. Histone methylation mainly consists of lysine and arginine methylation, which play an important role in controlling genomic stability, regulation of transcription and pluripotency of stem cells. Recent studies have found that histone methylation has a crucial role in early embryonic development and pluripotency maintenance of stem cell [25–28]. Aberrant methylation of H3R26 was maximal in four-cell blastomeres, which will contribute to development of ICM and influence on cell fate determination. This was the first evidence for abnormal epigenetic modification during early embryonic development can regulate developmental fate of blastomere [25]. Canovas et al. found that demethylation of H3K27me3 before and after embryonic genome activation to ensure normal bovine IVF embryonic development [27]. Methylation of histone at specific residue is one of the most important epigenetic modifications and has an essential role in both transcriptional activation and repression. Generally, H3K9, H3K27, H4K20 methylations mainly inhibit gene expression, whereas methylations of H3K4, H3K36, H3K79 are related with activation of gene transcription. Consistent with the histone acetylation, histone methylation is a reversible dynamic epigenetic modification, since most histone demethylase have been found except for H3K79 and H4K20.

Asymmetric distribution of H3K9me2/3 was detected in pronuclear stage murine SCNT embryos [29], whereas the data from pronuclear stage porcine embryos was contradictory [30]. Our study found neither IVF nor SCNT embryos showed asymmetric distribution of H3K9me2/3 at pronuclear stage. High expression of H3K9me2/3 was observed until 4-cell stage of SCNT embryos. No differences on H3K9me2 expression level were observed between the ICM and TE in SCNT blastocyst, but significantly higher than those in IVF counterparts, which could be due to the aberrant reprogramming of H3K9me2 in porcine SCNT embryos. H3K27me3 is an epigenetic modification involving in the maintenance of cellular pluripotency [26]. It was already proved that H3K27me3 was only expressed in ICM of mouse fertilized blastocysts [19] and also played an important role in genome activation of bovine fertilized embryos [27]. We found symmetric distribution pattern of H3K27me3 in pronuclear stage of porcine IVF and SCNT embryos. Although demethylation and remethylation occurred during development until blastocysts stage of SCNT embryos, the methylation level was lower and also lost asymmetric distribution pattern in ICM and TE of SCNT blastocyst. However, dynamic changes of H3K27me2 were similar in the whole developmental stage of SCNT and IVF embryos, which could indicate faithful reprogramming of H3K27me2 occur in porcine SCNT early embryos. H4K20me2/3, without specific demethylase, is primarily associated with transcriptional repression and genome stability [31–33]. Their roles in the developmental biology of early embryo or stem cells have not been reported. We found that H4K20me2/3 did not exhibit asymmetric distribution at pronuclear stage of IVF and SCNT embryos. The remethylation level of H4K20me2 in SCNT embryo was lower than that in IVF embryo during the development from morula to expanded blastocyst stage. On the contrary, retention of massive H4K20me3 modification of donor cell could hinder SCNT embryonic genome activation. Unlike fertilized blastocysts, SCNT blastocysts did not show asymmetric distribution of hypomethylation in ICM and hypermethylation in TE, which could cause developmental defect of lineage in SCNT blastocyst.

H3K4me2 is the first identified histone methylation, which can be initiatively demethylated. Studies have found that increase in H3K4me2 expression at 2-cell stage of mouse fertilized embryos causes abnormal activation of embryonic gene expression and further reduction of developmental efficiency [34]. SCNT mouse embryo showed lower H3K4me2 intensity than IVF embryos at 2-cell stage, and then significantly increased until the 8-cell stage. Abnormal expression of H3K4me2 was detected during genome activation and remethylation of mouse cloned embryo [35]. Despite hypermethylation of H3K4me3 from the 8-cell to the blastocysts stage, H3K4me3 expression is almost constant from pronuclear to the 4-cell stage between SCNT and IVF. Both IVF and SCNT blastocyst showed symmetric distribution in H3K4me2 expression, which is in contradiction with a previous study in the mouse, is probably due to evolutionary differences in species [35]. Covalent domain formed by H3K4me3 and H3K7me3 involved in the regulation of embryonic stem cell pluripotency. H3K4me3 expression level decrease and then increase in early development of human, mouse and porcine naturally fertilized embryos, and then symmetrically distributed in ICM and TE of blastocyst stage [23, 36]. H3K4me3 expression from 2- cell to 4- cell stage was significantly higher in mouse SCNT embryos than that in IVF embryos [19]. High level of H3K4me3 methylation was detected until 4-cell stage of SCNT embryos in the present study, which were probably due to the insufficient demethylation of H3K4me3 during porcine somatic cell reprogramming. Recently, Huang et al. found that increased expression of H3K4me3 at the 4-cell and blasotycst stages probably compromise pig SCNT embryo development by interferring balance of H3K4me3-H3K27me3 modifications [12]. H3K36me2/3 associated with transcriptional activation and cell proliferation. JHDM1A (also known as KDM2A) is an H3K36 demethylase that regulates cell proliferation [37]. Mouse somatic cell reprogramming efficiency does not depend on cell proliferation, but achieved by increase in early pluripotent gene expression during somatic cell reprogramming [38]. Similar with IVF embryo, we found no change in expression of H3K36me2 during early development of porcine somatic cell reprogramming process and absence of asymmetric distribution of lineages differentiation, which probably activate the cell proliferation or promote pluripotent gene expression during embryonic development. H3K36me3 expression initially decreases and then increases during early development of porcine SCNT embryos, which was similar to IVF embryo. These results indicated that no abnormality in H3K36me2/3 expression during porcine somatic cell reprogramming process, which is consistent with findings from Diao et al.[13] H3K79me2/3 is a special epigenetic modification located inside of core histone. Both H3K79me2 and H3K79me3 suddenly decreased soon after fertilization and remained undetectable from 2-cell to blastocyst stage, which suggested that H3K79 methylation plays an important role in the epigenetic reprogramming of mouse cloned embryo in earlier developmental period [39]. Recent study found specifically knock down the H3K79 histone methyltransferase DOT1L by shRNA significantly increased the expression of *Nanog* and *Lin28*, which finally enhanced 3- or 4-fold of mouse reprogramming efficiency [40]. We found high expression level of H3K79me2/3 in porcine MII oocytes, which can be quickly removed by oocyte cytoplasm after fertilization. The symmetric distribution pattern of H3K79me2 was detected in both ICM and TE lineages in blastocysts, whereas H3K79me3 remained undetectable. These results suggested special expression pattern of H3K79me2/3 in early embryos might accelerate reprogramming progression of somatic genome and promote the expression of some important genes associated with lineage development.

**Fig 7:**
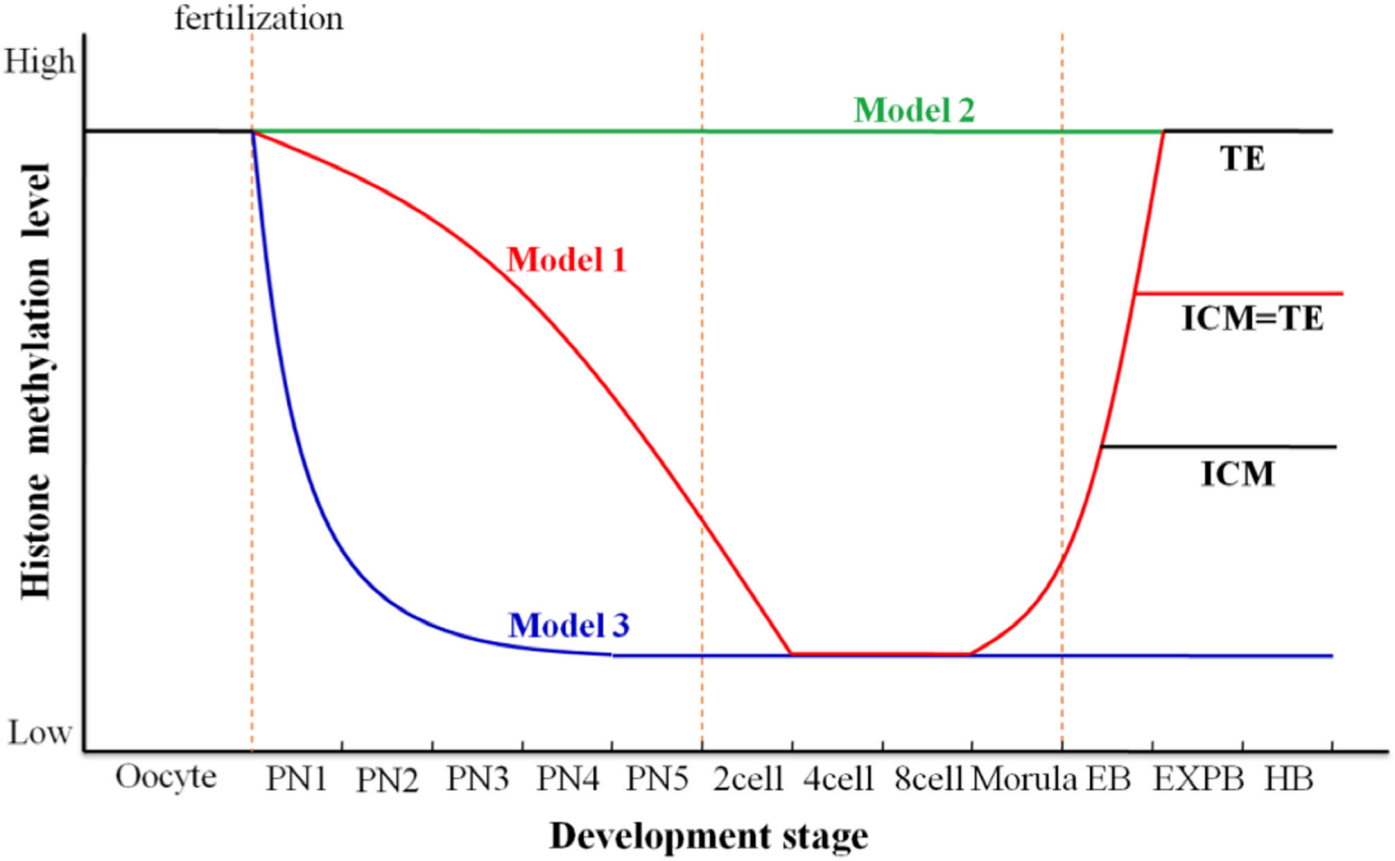
Dynamic reprogramming model for histone lysine methylation during porcine early embryo development. We propose the following three distinct models about dynamic reprogramming of histone lysine methylation during porcine early embryo development. Red line indicates the first model, the signal intensity from the majority of histone lysine methylation modifications (H3K4me2/me3, H3K9me2/me3, H3K27me2/me3, H3K36me3, and H4K20me2/me3) gradually decrease from pronuclear stage to 8-cell stage and reappear at morula stage. Histone methylations at blastocyst stage show symmetric distribution pattern with similar methylation level between both cell lineages or asymmetric distribution pattern as hypermethylation in TE and hypomethylation in ICM. Green line indicates the second model, the expression level of histone methylation (H3K36me2) invariably keep constant from pronuclear stage to blastocyst stage and the signal intensity is almost consistent between both cell lineages. Blue line indicates the third model, histone methylation (H3K79me2 and H3K79me3) is quickly demethylated after fertilization and always keep low level during development to the blastocyst stage.

In summary, H3K9me2, H3K9me3, H4K20me3 and H3K4me3 have not been fully removed until the 4-cell of porcine SCNT embryo, which could lead to unsuccessful activation of the embryonic genome and insufficient synthesis of regulators required for late development, finally result in low developmental efficiency of cloned embryo. On the other hand, abnormally high expression of H3K9me2, H3K9me3, H3K27me3 and H4K20me3 in TE lineage cells could cause aberrant gene expression of trophectodermal derivative (placenta) and developmental defects, and ultimately result in abortion or death of SCNT embryos at different developmental stages. Therefore, we propose the following three distinct models on dynamic reprogramming of histone lysine methylation during porcine early embryo development (Fig 7). The first model, the signal intensity from the majority of histone lysine methylation modifications (H3K4me2/3, H3K9me2/3, H3K27me2/3, H3K36me3, and H4K20me2/3) gradually decrease from pronuclear stage to 8-cell stage and reappear at morula stage. These histone methylations at blastocyst stage show symmetric distribution pattern with similar methylation level between both cell lineages or asymmetric distribution pattern as hypermethylation in TE and hypomethylation in ICM. The second model, the expression level of histone methylation (H3K36me2) invariably keeps constant from pronuclear stage to blastocyst stage and the signal intensity is almost identical between both cell lineages. The third model, histone methylation is quickly demethylated after fertilization and always keeps low expression until 8-cell (H3K79me3) or blastocyst stage (H3K79me2).

Taken together, global histone methylation modifications can be reprogrammed correctly through three different programs in porcine IVF early embryos. However, histone methylations show stage-specific abnormal distribution patterns during early development of porcine SCNT embryos. Therefore, we could modify aberrant targets of histone methylation at early stage to improve cloning efficiency in the near future.

## Supporting Information

S1 Fig Related with Fig 1. Dynamic patterns of H3K4me2/3 during pronuclear embryo development. **(A)** Representative images of porcine IVF and SCNT pronuclear embryos at different developmental stages immunostained with an anti-H3K4me2 antibody. Antibody was localized with an Alexa Flour 488-conjugated secondary antibody (green). DNA was stained with propidium iodide (red). Middle panels showed the merged images (yellow) between H3K4me2 signal (green) and DNA staining (red). **(B)** Representative images of porcine IVF and SCNT pronuclear embryos at different developmental stages immunostained with an anti-H3K4me3 antibody. Scale bar = 50 μm.
(TIF)

S2 Fig Related with Fig 2. Expression patterns of H3K9me2/3 in pronuclear embryo. **(A)** Representative images of porcine IVF and SCNT pronuclear embryos at 16–18 hpi (hpa) stage immunostained with an anti-H3K9me2/me3 antibody. Antibody was localized with an Alexa Flour 488-conjugated secondary antibody (green). DNA was stained with propidium iodide (red). Middle panels showed the merged images (yellow) between H3K9me2/me3 signal (green) and DNA staining (red). ♂, male pronucleus. ♀, female pronucleus. Scale bar = 50 μm.
(TIF)

S3 Fig Related with Fig 3. Expression patterns of H3K27me2/3 in pronuclear embryo. **(A)** Representative images of porcine IVF and SCNT pronuclear embryos at 16–18 hpi (hpa) stage immunostained with an anti-H3K27me2/me3 antibody. Antibody was localized with an Alexa Flour 488-conjugated secondary antibody (green). DNA was stained with propidium iodide (red). Middle panels showed the merged images (yellow) between H3K27me2/me3 signal (green) and DNA staining (red). ♂, male pronucleus. ♀, female pronucleus. Scale bar = 50 μm.
(TIF)

S4 Fig Related with Fig 4. Expression patterns of H3K36me2/3 in pronuclear embryo. **(A)** Representative images of porcine IVF and SCNT pronuclear embryos at 16–18 hpi (hpa) stage immunostained with an anti-H3K36me2/me3 antibody. Antibody was localized with an Alexa Flour 488-conjugated secondary antibody (green). DNA was stained with propidium iodide (red). Middle panels showed the merged images (yellow) between H3K36me2/me3 signal (green) and DNA staining (red). ♂, male pronucleus. ♀, female pronucleus. Scale bar = 50 μm.
(TIF)

S5 Fig Related with Fig 5. Expression patterns of H3K79me2/3 in MII oocyte and pronuclear embryo. **(A)** Representative images of porcine MII oocytes immunostained with an anti-H3K79me2/me3 antibody. Representative images of porcine IVF and SCNT pronuclear embryos immunostained with an anti-H3K79me2 antibody. **(B)** Representative images of porcine IVF and SCNT pronuclear embryos immunostained with an anti-H3K79me3 antibody. Scale bar = 50 μm.
(TIF)

S6 Fig Related with Fig 6. Expression patterns of H4K20me2/3 in pronuclear embryo. **(A)** Representative images of porcine IVF and SCNT pronuclear embryos at 16–18 hpi (hpa) stage immunostained with an anti-H4K20me2/me3 antibody. Antibody was localized with an Alexa Flour 488-conjugated secondary antibody (green). DNA was stained with propidium iodide (red). Middle panels showed the merged images (yellow) between H4K20me2/me3 signal (green) and DNA staining (red). ♂, male pronucleus. ♀, female pronucleus. Scale bar = 50 μm.
(TIF)

S1 Table Antibodies. (DOC)
