# Supplementary figures and images for: Genome-Wide Dynamic Profiling of Histone Methylation during Nuclear Transfer-Mediated Porcine Somatic Cell Reprogramming
Source: PLoS One. 2015 Dec 18;10(12):e0144897. doi: 10.1371/journal.pone.0144897 (PMC4687693; doi:10.1371/journal.pone.0144897)

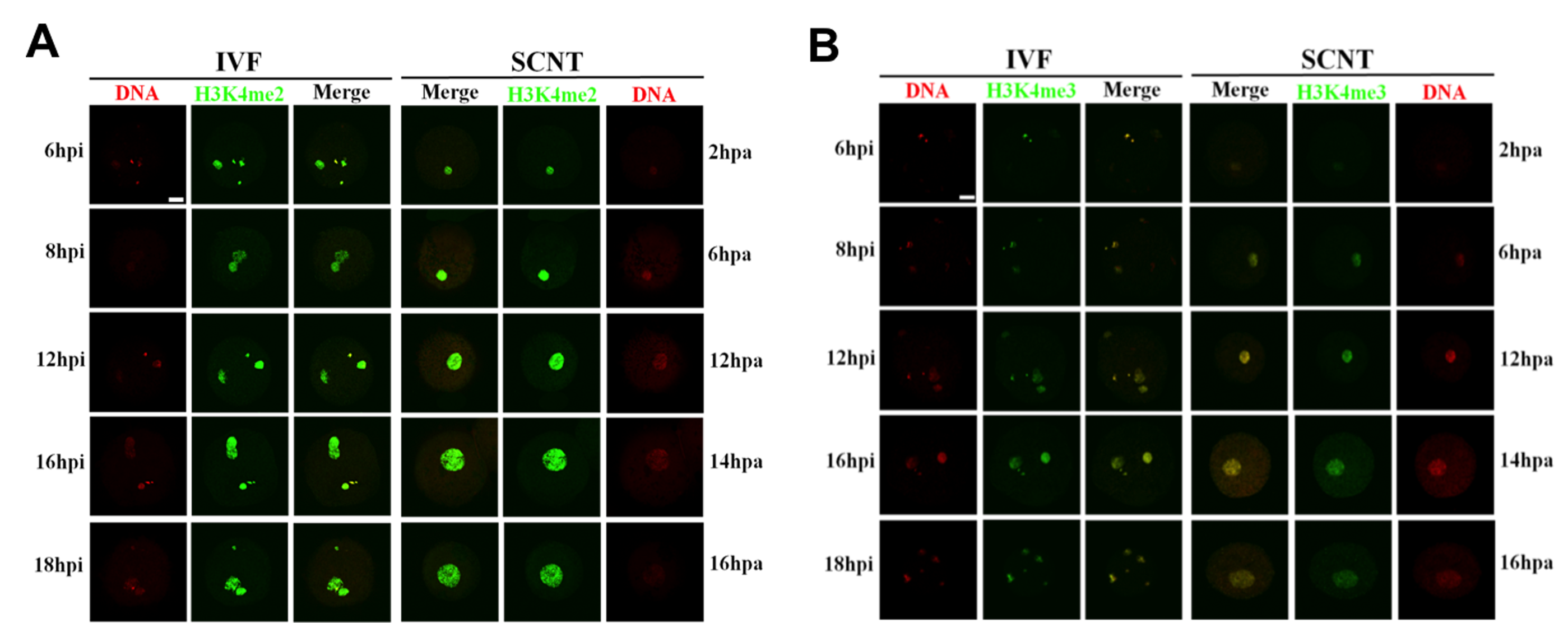

Supplement: S1 Fig — (A) Representative images of porcine IVF and SCNT pronuclear embryos at different developmental stages immunostained with an anti-H3K4me2 antibody. Antibody was localized with an Alexa Flour 488-conjugated secondary antibody (green). DNA was stained with propidium iodide (red). Middle panels showed the merged images (yellow) between H3K4me2 signal (green) and DNA staining (red). (B) Representative images of porcine IVF and SCNT pronuclear embryos at different developmental stages immunostained with an anti-H3K4me3 antibody. Scale bar = 50 μm. (TIF) [file pone.0144897.s001.tif]

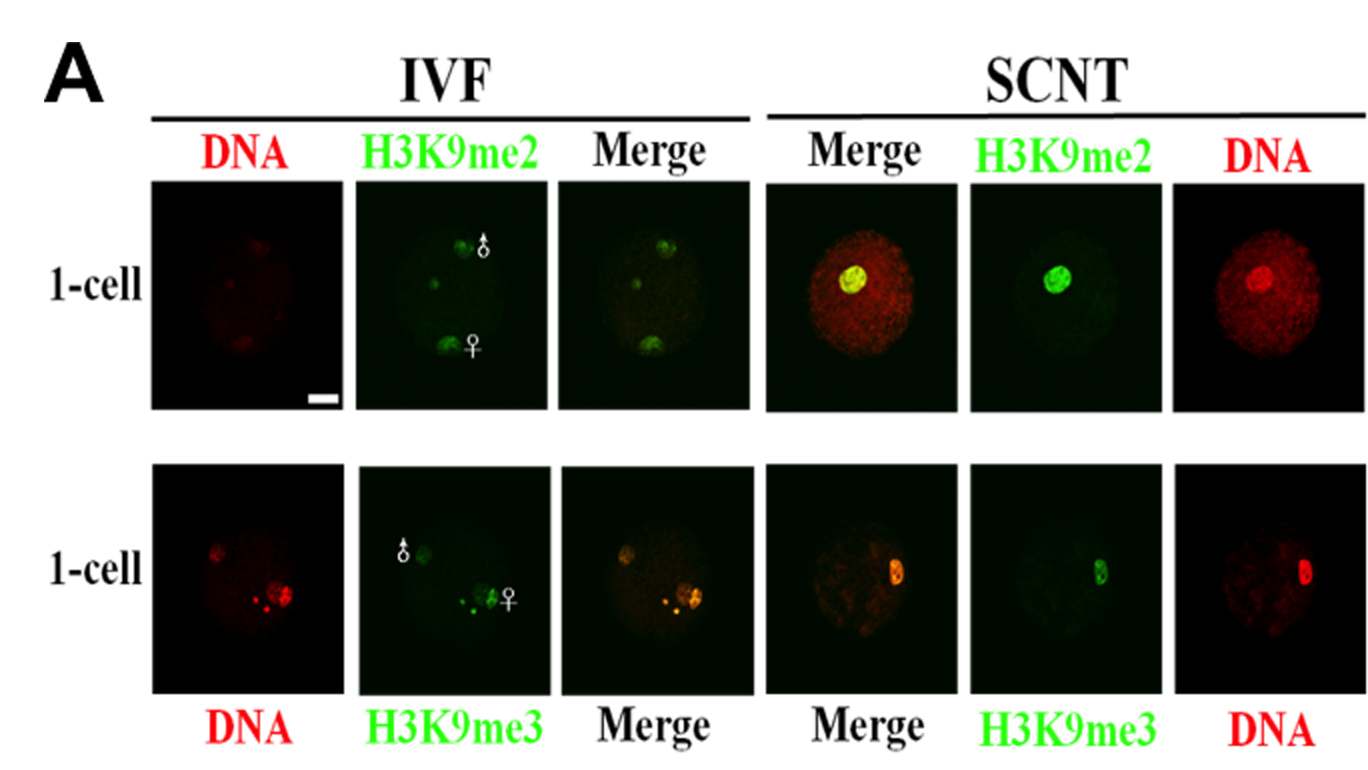

Supplement: S2 Fig — (A) Representative images of porcine IVF and SCNT pronuclear embryos at 16–18 hpi (hpa) stage immunostained with an anti-H3K9me2/me3 antibody. Antibody was localized with an Alexa Flour 488-conjugated secondary antibody (green). DNA was stained with propidium iodide (red). Middle panels showed the merged images (yellow) between H3K9me2/me3 signal (green) and DNA staining (red). ♂, male pronucleus. ♀, female pronucleus. Scale bar = 50 μm. (TIF) [file pone.0144897.s002.tif]

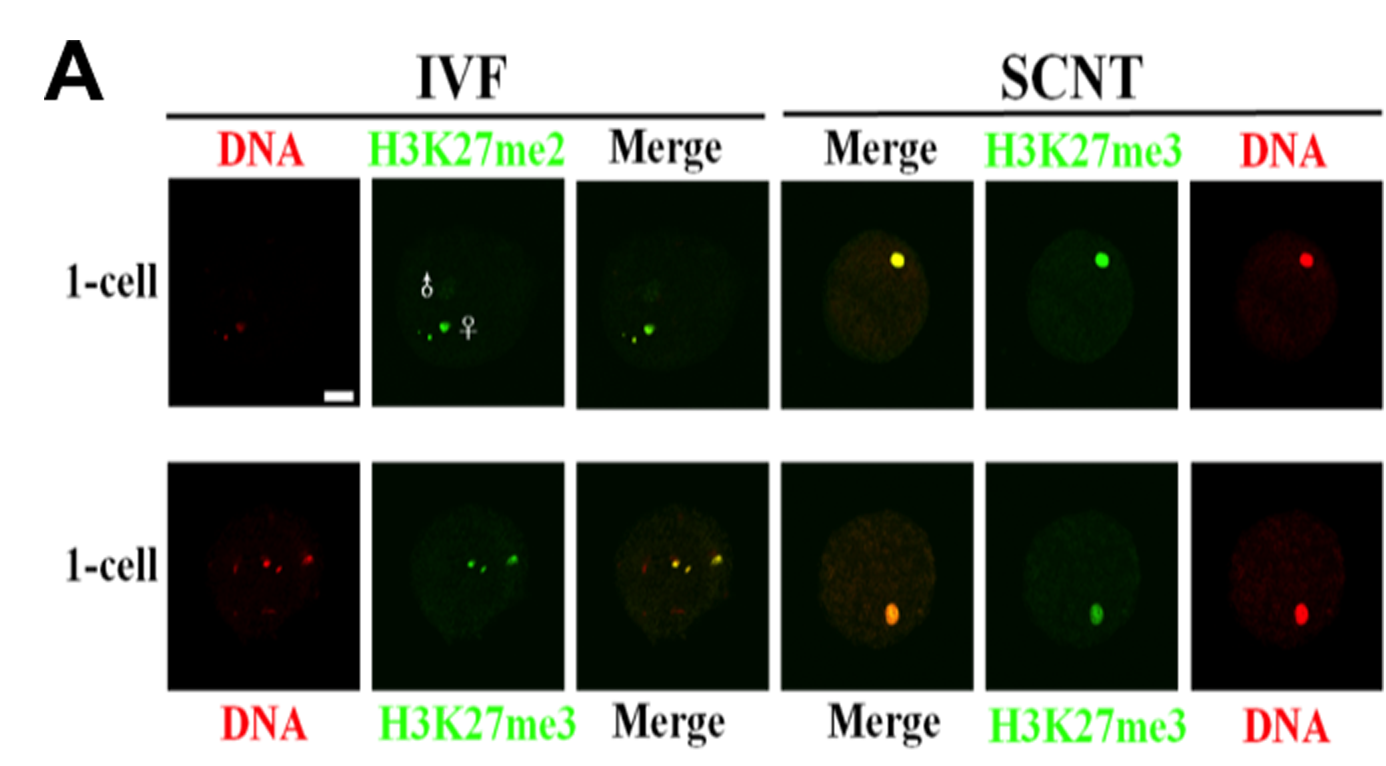

Supplement: S3 Fig — (A) Representative images of porcine IVF and SCNT pronuclear embryos at 16–18 hpi (hpa) stage immunostained with an anti-H3K27me2/me3 antibody. Antibody was localized with an Alexa Flour 488-conjugated secondary antibody (green). DNA was stained with propidium iodide (red). Middle panels showed the merged images (yellow) between H3K27me2/me3 signal (green) and DNA staining (red). ♂, male pronucleus. ♀, female pronucleus. Scale bar = 50 μm. (TIF) [file pone.0144897.s003.tif]

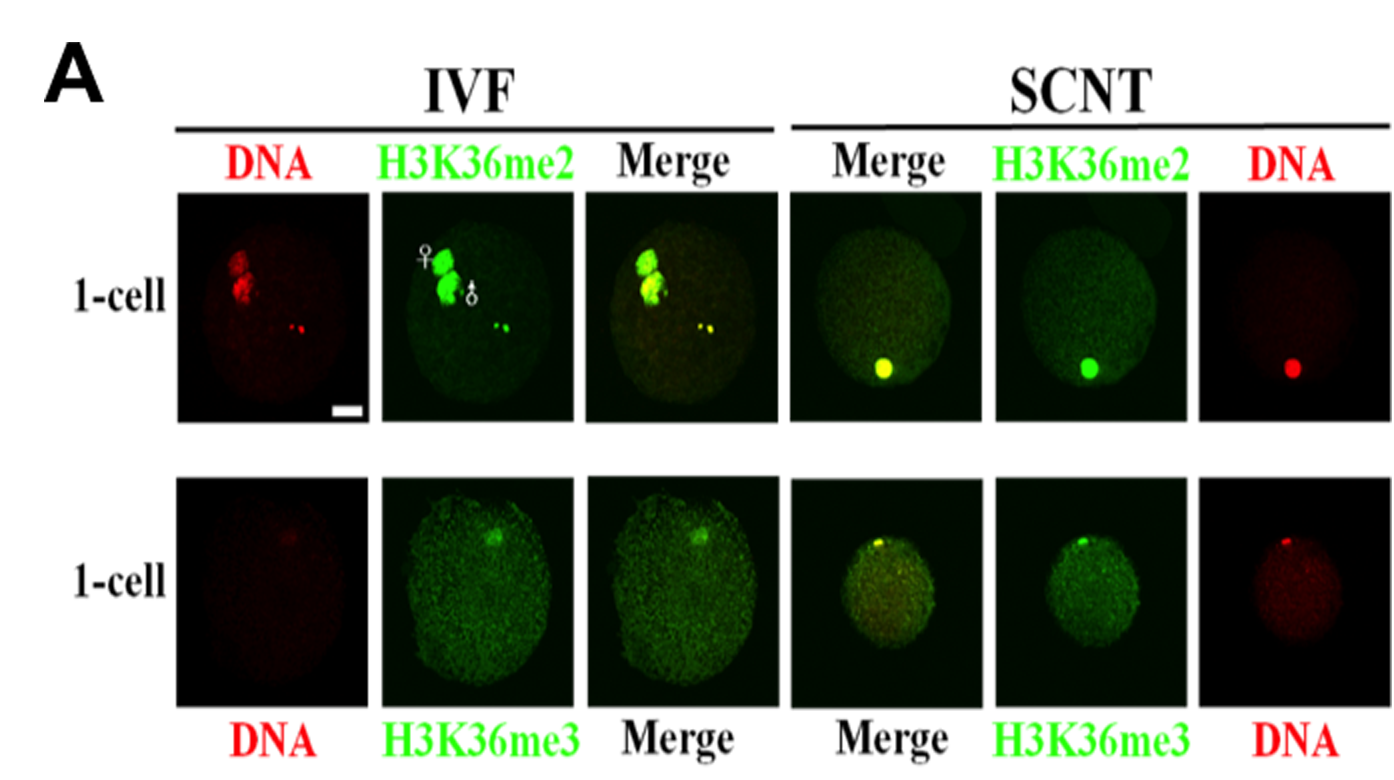

Supplement: S4 Fig — (A) Representative images of porcine IVF and SCNT pronuclear embryos at 16–18 hpi (hpa) stage immunostained with an anti-H3K36me2/me3 antibody. Antibody was localized with an Alexa Flour 488-conjugated secondary antibody (green). DNA was stained with propidium iodide (red). Middle panels showed the merged images (yellow) between H3K36me2/me3 signal (green) and DNA staining (red). ♂, male pronucleus. ♀, female pronucleus. Scale bar = 50 μm. (TIF) [file pone.0144897.s004.tif]

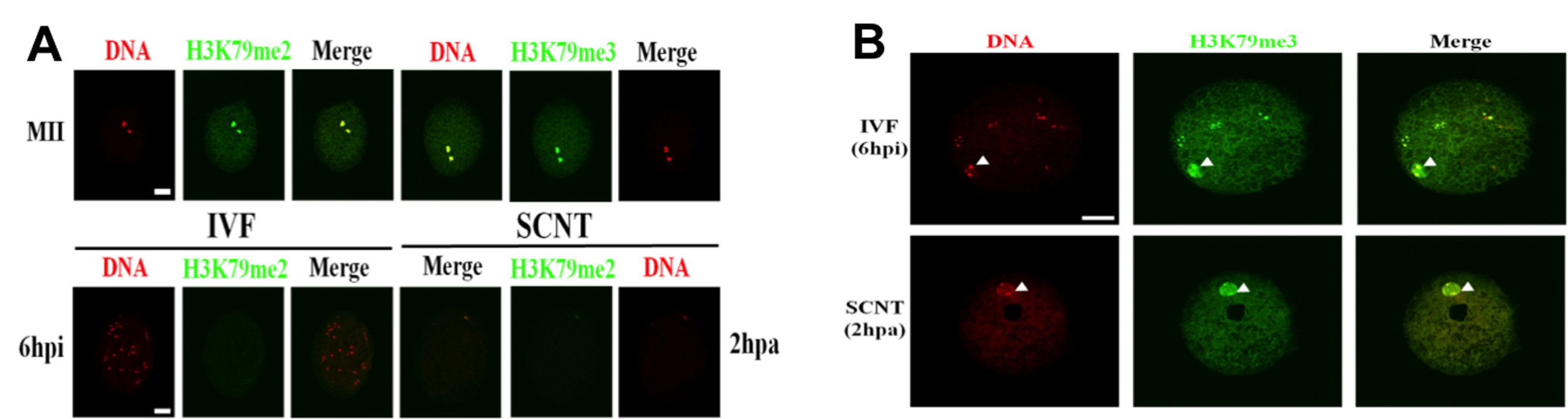

Supplement: S5 Fig — (A) Representative images of porcine MII oocytes immunostained with an anti-H3K79me2/me3 antibody. Representative images of porcine IVF and SCNT pronuclear embryos immunostained with an anti-H3K79me2 antibody. (B) Representative images of porcine IVF and SCNT pronuclear embryos immunostained with an anti-H3K79me3 antibody. Scale bar = 50 μm. (TIF) [file pone.0144897.s005.tif]

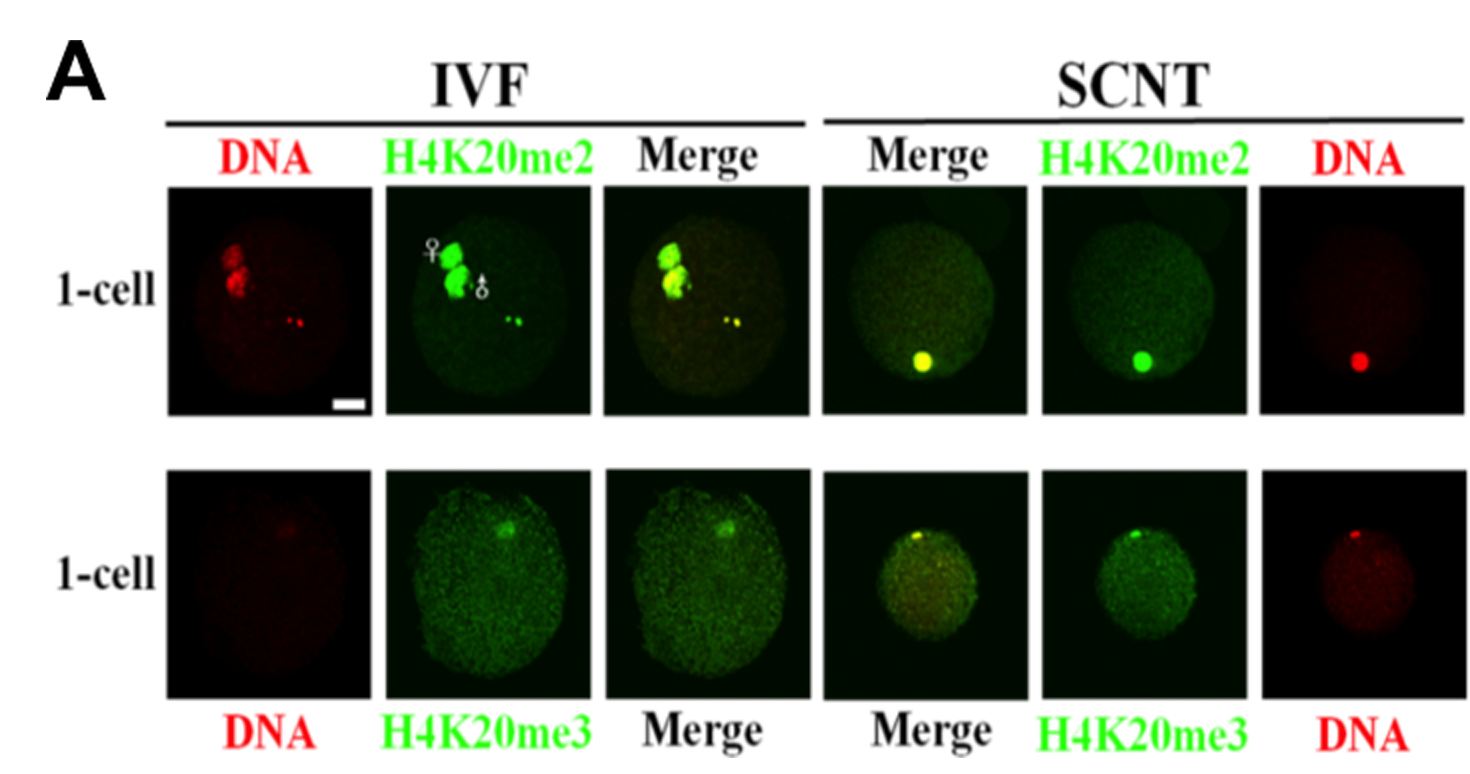

Supplement: S6 Fig — (A) Representative images of porcine IVF and SCNT pronuclear embryos at 16–18 hpi (hpa) stage immunostained with an anti-H4K20me2/me3 antibody. Antibody was localized with an Alexa Flour 488-conjugated secondary antibody (green). DNA was stained with propidium iodide (red). Middle panels showed the merged images (yellow) between H4K20me2/me3 signal (green) and DNA staining (red). ♂, male pronucleus. ♀, female pronucleus. Scale bar = 50 μm. (TIF) [file pone.0144897.s006.tif]
