# Supplementary material for: Genome-Wide Dynamic Profiling of Histone Methylation during Nuclear Transfer-Mediated Porcine Somatic Cell Reprogramming
Source: PLoS One. 2015 Dec 18;10(12):e0144897. doi: 10.1371/journal.pone.0144897 (PMC4687693; doi:10.1371/journal.pone.0144897)
Supplement: S1 Table — (DOC) [file pone.0144897.s007.doc]

Table S1. antibodies

| **Primary antibody** | **Species** | **Vendor** | **Cat.no. and Dilution** |
| --- | --- | --- | --- |
| **H3K4me2** | **Rabbit** | **Abcam** | **ab32356 1:200** |
| **H3K4me3** | **Rabbit** | **Abcam** | **ab8580 1:200** |
| **H3K9me2** | **Mouse** | **Abcam** | **ab1220 1:200** |
| **H3K9me3** | **Rabbit** | **Abcam** | **ab8898 1:200** |
| **H3K27me2** | **Rabbit** | **Abcam** | **ab24684 1:200** |
| **H3K27me3** | **Rabbit** | **Upstate** | **07-449 1:200** |
| **H3K36me2** | **Rabbit** | **Abcam** | **ab9049 1:200** |
| **H3K36me3** | **Rabbit** | **Abcam** | **ab9050 1:200** |
| **H3K79me2** | **Rabbit** | **Abcam** | **ab3594 1:200** |
| **H3K79me3** | **Rabbit** | **Abcam** | **ab2621 1:200** |
| **H4K20me2** | **Rabbit** | **Abcam** | **ab9052 1:200** |
| **H4K20me3** | **Rabbit** | **Abcam** | **ab9053 1:200** |
